# Relationships between body image, dyadic coping and post-traumatic growth in breast cancer patients: a cross-sectional study

**DOI:** 10.3389/fpsyg.2024.1368429

**Published:** 2024-05-09

**Authors:** Yuan Wang, Shan Wang, Ling Tong, Jiaru Zhuang, Yihan Xu, Yibo Wu, Ling Chen

**Affiliations:** ^1^Human Reproductive and Genetic Center, Affiliated Hospital of Jiangnan University, Jiangsu, China; ^2^Shanghai Medical College of Fudan University, Shanghai, China; ^3^Department of Breast Surgery, Affiliated Hospital of Jiangnan University, Jiangsu, China

**Keywords:** cross-sectional study, breast cancer, nursing, body image, dyadic coping, posttraumatic growth

## Abstract

**Background:**

The diagnosis and treatment of cancer triggers not only a negative psychological response for the patient, but also a positive psychological outcome. Positive dyadic coping, as a form of coping for mental health outcomes, can maintain or reestablish internal stability between the patient and his or her spouse, resulting in positive physical and psychological changes. However, there is a paucity of research on body image, dyadic coping, and post-traumatic growth in breast cancer patients. The purpose of this study was to explore the relationship and pathways between body image, dyadic coping, and post-traumatic growth in breast cancer patients.

**Methods:**

A cross-sectional study was conducted from November 2022 to November 2023 at a tertiary care hospital in Wuxi, Jiangsu, China. This study was conducted among 154 breast cancer patients treated at the Affiliated Hospital of Jiangnan University, all of whom completed demographic and clinical information questionnaires, Body image self-rating questionnaire for breast cancer (BISQ-BC), Dyadic Coping Inventory (DCI) and Post Traumatic Growth Inventory (PTGI). A Pearson correlation analysis was used to explore the relationship between body image, dyadic coping, and post-traumatic growth. Structural equation modeling was used to analyze the path relationships among the three and to explore the mediating role of dyadic coping.

**Results:**

The level of body image was negatively correlated with post-traumatic growth (*r* = −0.462, *p* < 0.01); and the level of body image was negatively correlated with dyadic coping (*r* = −0.308, *p* < 0.01). And dyadic coping was positively associated with post-traumatic growth (*r* = 0.464, *p* < 0.01). The structural equation modeling results supported the mediation model with the following model fit indices, chi-square to degrees of freedom ratio (χ^2^/df = 2.05), goodness of fit index (GFI = 0.93), comparative fit index (CFI = 0.99), canonical fit index (NFI = 0.93), incremental fit index (IFI = 0.99), non-canonical fit index (TLI = 0.99) and the root mean square of the difference in approximation error (RMSEA = 0.03). Body image and dyadic coping directly affected post-traumatic growth (*β* = −0.33, *p* < 0.05; *β* = 0.43, *p* < 0.05). And body image indirectly influenced post-traumatic growth through dyadic coping (*β* = −0.17, *p* < 0.05).

**Conclusion:**

Interconnections between body image, dyadic coping, and post-traumatic growth in breast cancer patients. A preliminary validation of the mediating role of dyadic coping between body image and post-traumatic growth, body image can have an impact on dyadic coping, which in turn can have an impact on post-traumatic growth. Whereby higher levels of dyadic coping in patients may also be associated with higher levels of post-traumatic growth, whereas body image disturbance may impede levels of post-traumatic growth.

## 1 Introduction

According to the latest data from the International Agency for Research on Cancer (IARC), the number of new breast cancer patients worldwide reached 2.26 million in 2020 (Ferlay et al., 2021). Of these, more than 1.1 million women are newly diagnosed with breast cancer globally each year, and 410,000 women die from the disease (Faroughi et al., 2023). And Chinese women with breast cancer account for about 18% of global BC deaths (Cao et al., 2018). At this stage, treatment options for breast cancer include surgery, chemotherapy, radiotherapy, endocrinology, targeting, and so on (Zeng et al., 2018). These treatment modalities bring many negative effects to patients while fighting tumors (Britt et al., 2020), leading to serious physical, physiological, and psychological damage (Trapani et al., 2022; Wilkinson and Gathani, 2022). Surgical treatment leaves patients with partial or total loss of breasts, and the integrity of the female body is destroyed, causing many female patients to believe that they have lost their female symbols, affecting their unique female charms, and thus they are unwilling to look at their own bodies directly (Paterson et al., 2016). Scarring, swelling, redness of the skin, and lymphedema also occur after surgery. Chemotherapy causes patients to lose their hair, as well as weight gain and facial edema. Radiation therapy leads to permanent localized skin hyperpigmentation, dryness, burns, small and hardened breasts on the affected side, and edematous fibrosis of the breasts (Collins et al., 2011). Endocrine therapy causes patients to experience menopause-like symptoms, body aches, and decreased libido. Targeted therapy causes patients to experience skin changes such as flaking, itching, and nail hyperpigmentation. All these problems can lead to body image disturbance (BID) in breast cancer patients (Afshar-Bakshloo et al., 2023).

Body image disturbance can increase the psychological burden of patients, causing them to experience negative emotions such as disease shame, anxiety and depression, which can affect their mental health and even increase their risk of death (Alhusban, 2019). Scholars such as Trindade et al. (2018) have argued that poorer body image increases psychological distress in patients. However, more current research has focused on the association between body image and negative psychological outcomes, and very little has examined the impact of body image on patients’ post-traumatic growth. The results of the study by Suyi et al. (2018) showed that good body image in breast cancer patients helped to improve their post-traumatic growth. With the development of positive psychology, research has shown that cancer patients not only have negative emotional experiences in the process of coping with emergencies, but also positive psychological changes, i.e., post-traumatic growth (Bi et al., 2021; Shi et al., 2021). Post-traumatic growth is not produced by the disease itself, but rather during a patient’s experience of trauma, when the body and mind process and react to the event in an integrated manner. Therefore, when a patient acquires a “new body,” how to face and accept this “new body” is crucial to the development of post-traumatic growth (Su and Fang, 2018). Increasing the level of post-traumatic growth in breast cancer patients enables them to maintain a healthy lifestyle, such as regular exercise and a sensible diet, which contributes to a better quality of life (Tomita et al., 2017).

Dyadic coping refers to the joint response of the patient and his or her partner as a whole in response to a stressful event, when the patient expresses stress to his or her partner and the partner receives the stress signal (Hu et al., 2022). The Systemic Transactional Model (STM) states that a dyadic coping process can be triggered when a stressor communicates stress to his or her partner verbally, emotionally, or physically, and the partner receives and interprets the stress signal and responds with some type of coping (Bodenmann et al., 2006; Bodenmann and Randall, 2012). In addition, STM states that positive dyadic coping can maintain or re-establish internal stability between the patient and his or her spouse, strengthening communication, cohesion, and relationship satisfaction of both spouses, thus promoting positive physiological and psychological changes in the patient (Gesselman et al., 2017). The results of the study by Zhang et al. (2019) showed that a dyadic coping intervention was effective in promoting the level of post-traumatic growth in elderly stroke patients. Therefore, STM was chosen as the theoretical framework for this study.

Treatment for breast cancer can improve survival outcomes for patients, and as treatment outcomes and survival rates improve, the importance of assessing post-treatment sequelae becomes more important (Li, 2022). Because treatment can lead to appearance-related side effects (e.g., loss of breast, scarring, hair loss, weight gain, muscle loss, etc.), these side effects have been used as a contributing factor in the development of a negative body image in women diagnosed with cancer (Brunet et al., 2013). Therefore, an emphasis on body image is necessary. However, a BC diagnosis can elicit not only negative reactions, but also positive psychological outcomes, i.e., beneficial outcomes through struggling with the traumatic event (Li, 2022). Post-traumatic growth is defined as positive cognitive and affective changes experienced as a result of struggling with a traumatic event (Ning et al., 2023). Interference with body image has been recognized as a barrier to positive factors (Kling et al., 2019). However, Hefferon et al. (2009) reviewed that the body is an important part of the process and outcome of post-traumatic growth based on open-ended interviews with 10 British Columbia survivors. Further research is needed on the relationship between body image and psychological outcomes, particularly positive outcomes. Previous research has shown that dyadic coping can be used as a form of coping for mental health outcomes, and that through positive dyadic coping, it can promote post-traumatic growth in patients, and that spouses play a vital role in the coping process of individual cancer patients with the disease (Zhang et al., 2022). However, to date, there have been no studies examining the relationship between the body image, dyadic coping, and post-traumatic growth in breast cancer patients.

**Figure 1:**
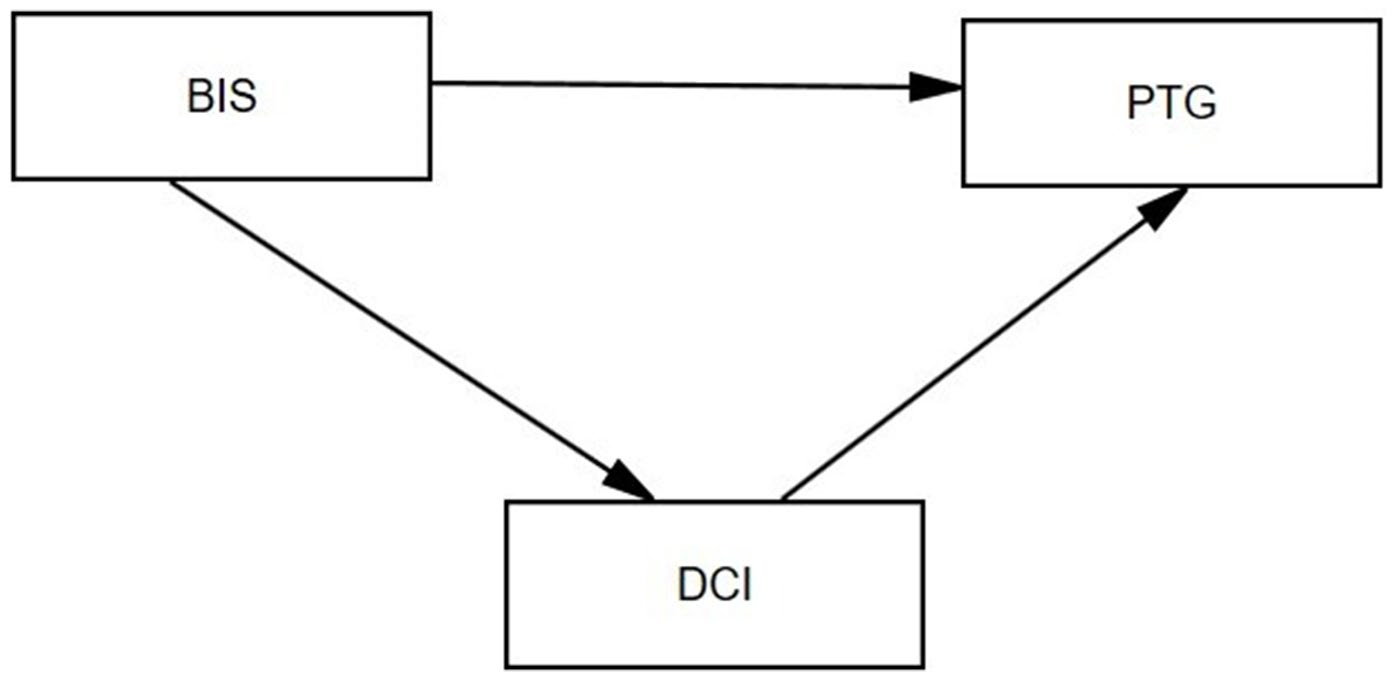
The hypothesis model of body image, dyadic coping, and post-traumatic growth (PTG) in patients with breast cancer. BIS: Body image self-rating. DCI: dyadic coping inventory. PTG: post-traumatic growth.

Previous studies have shown that poor level of body image in breast cancer patients can hinder their post-traumatic growth (Su and Fang, 2018). Positive dyadic coping style can improve post-traumatic growth in stroke patients (Zhang et al., 2022). STM divided dyadic coping into positive coping (stress communication, support coping, delegated coping and common coping) and negative coping (contradictions and hostility). In addition, stress communication is an important part of dyadic coping, which refers to couples openly expressing their thoughts and feelings about the disease (Bodenmann et al., 2019). However, the research results of An et al. (2018) found that breast cancer patients were in the status quo of low body image level, so patients with low body image level generated negative psychological feelings such as inferiority and shame, which made them reluctant to share their thoughts and feelings with their husbands, resulting in estrangement and suspicion between the two sides, thus impeding the dyadic behavior of the couple. Therefore, this study created a structural equation modeling diagram based on STM as a theoretical basis (see Figure 1) and made two hypotheses: (1) There is a correlation between the body image, dyadic coping and post-traumatic growth in breast cancer patients: the level of body image is negatively correlated with dyadic coping and post-traumatic growth, while dyadic coping is positively correlated with post-traumatic growth. (2) Body image indirectly affects post-traumatic growth through dyadic coping: body image can have an impact on dyadic coping, which in turn can have an impact on post-traumatic growth.

## 2 Materials and methods

### 2.1 Ethical recognition

The study design was approved by the Ethics Committee of Affiliated Hospital of Jiangnan University (JNMU020423785). Each participant signed an informed consent form, and patient privacy was protected throughout the study.

### 2.2 Participants and measures

This cross-sectional study was conducted between November 2022 and November 2023 in postoperative breast cancer patients undergoing active treatment (radiotherapy, chemotherapy, hormone therapy, etc.). A convenience sampling method was used to recruit subjects. All subjects were recruited from the Department of Breast Surgery, Affiliated Hospital of Jiangnan University, Wuxi, Jiangsu, China, and the inclusion criteria were as follows: (1) age ≥ 18; (2) pathologic diagnosis of breast cancer; (3) surgical treatment; (4) all patients were married women and lived with their spouses; and (5) were conscious, with good reading and comprehension skills. Exclusion criteria: (1) received psychotherapy; (2) associated with other malignancies or severe cardiac, hepatic, and renal dysfunction. The study subjects were selected based on strict inclusion and exclusion criteria. The purpose, significance and content of the survey were explained to each patient prior to the survey. Uniformly trained investigators used a uniform questionnaire in the survey and surveyed participants’ information through face-to-face methods.

### 2.3 Research instruments

Four instruments were used in this study: demographic and clinical information questionnaires, Body image self-rating questionnaire for breast cancer (BISQ-BC), Dyadic Coping Inventory (DCI) and Post Traumatic Growth Inventory (PTGI).

1. Demographic and Clinical Information Questionnaire

The Demographic and Clinical Information Questionnaire was designed by the researchers and consisted of two parts: demographic information and clinical information. It included questions such as: age, education level, age of marriage, work status, religion, tumor stage, *per capita* monthly household income, current treatment received and duration of illness.

1. Body image self-rating questionnaire for breast cancer (BISQ-BC)

The body image self-rating questionnaire for breast cancer (BISQ-BC) revised by An et al. (2018) was used to assess the patients’ body image status. The BISQ-BC was compiled by Chinese scholar Kaina Zhou et al. in 2018. The revised version has 5 dimensions and 26 entries, including body image-related behavioral changes (7 entries), body image-related sexual activity (4 entries), body image-related role changes (5 entries), body image-related psychological changes (8 entries), and body image-related social changes (2 entries), and the entries are scored using a Likert 5-point scale, with a total score ranging from 26 to 130, with higher scores indicating a greater degree of body image disturbance in the patient. In this study, the Cronbach’s alpha for this scale was calculated to be 0.797.

1. Dyadic Coping Inventory (DCI)

Developed by Bodenmann et al. (2019) in 2008, Chineseized and cross-culturally adapted by Xu et al. (2016) in 2016. Prof. Bodenman also divided dyadic coping into negative and positively dyadic coping, which is used to measure couples’ shared reactions and coping styles in response to stressful events. The scale consists of 37 items divided into five dimensions: stress communication (8 entries), supportive coping (10 entries), empowering coping (4 entries), negative coping (8 entries), and common coping (5 entries). The last two entries are an overall evaluation of coping styles and are not included in the total score. Each item was scored on a 5-point Likert scale (1 = “rarely” to 5 = “often”). Higher scores indicate a more positive dyadic coping style. For this study, a Cronbach’s alpha of 0.765 was calculated for this scale.

1. Post-Traumatic Growth Inventory (PTGI)

The scale was developed by Tedeschi and Calhoun (1996) in 1996. Using the Post-Traumatic Growth Inventory developed by scholars such as Wang et al. (2011) in 2011. The scale mainly evaluates the level of positive changes in post-traumatic breast cancer patients, which mainly consists of 5 dimensions of relating to others, new possibilities, personal strength, spiritual change, and appreciation of life, with a total of 20 entries. The scale is rated on a 0–5 Likert 6 scale with a total score of 0–100, with higher scores representing better levels of post-traumatic growth. The Cronbach’s alpha coefficient in this study was 0.809.

## 3 Statistical analysis

SPSS 26.0 software and AMOS 24.0 software were used for statistical analysis. Frequencies and percentages were used as descriptive statistics for demographic and clinical variables. Data that followed a normal distribution were expressed as mean and standard deviation, otherwise, they were expressed as median (interquartile spacing). Since the body image, dyadic coping, and post-traumatic growth followed a normal distribution, the Pearson correlation test was chosen to analyze the relationship between the three. AMOS 24.0 software was used to construct a structural equation model to analyze the pathways of dyadic coping and body image on post-traumatic growth, using body image as the independent variable, dyadic coping as the mediator variable, and post-traumatic growth as the dependent variable. Model fit was assessed by testing the ratio of chi-square to degrees of freedom (χ^2^/df), goodness-of-fit index (GFI), comparative fit index (CFI), canonical fit index (NFI), incremental fit index (IFI), non-canonical fit index (TLI), and root mean square of the difference of the approximation error (RMSEA). Finally, the mediation effect test was performed using the bias-corrected percentile Bootstrap, with confidence intervals set at 95%, and confidence intervals that did not contain 0 indicated a significant mediation effect value. A two-sided test level was used, with *p* < 0.05 indicating a statistically significant difference.

## 4 Results

### 4.1 Socio-demographic characteristics of participants

Of the 154 breast cancer patients, the mean (SD) age was 49.10 years (range 28–62). 49.4% of the patients had been married for more than 10–30 years; 28.6% of the patients were at the high school or post-secondary level of education; 92.2% of the patients reported having no religious affiliation. And 40.9% of the patients had *per capita* monthly household income of the average economic level (RMB 2,000~5,000 per month). There were 33 (21.4%) patients in stage I, 72 (46.1%) in stage II, 45 (29.2%) in stage III, and 5 (3.2%) in stage IV. Other participant characteristics are shown in Table 1.

**Table 1:** Demographic characteristics of the patients with breast cancer (*n* = 154).

Variable	Frequency (*n*)	Percentage (%)
**Age (years)**		
≤30	3	1.9
31~40	16	10.4
41~50	54	35.1
≥51	81	52.6
**Education**		
Primary school	22	14.3
Junior high school	40	26.0
High/secondary school	44	28.6
Junior college	20	13
Bachelor and above	28	18.2
**Marriage length (years)**		
<10	13	8.4
10–30	76	49.4
>30	65	42.2
**Occupation**		
Incumbency	50	32.5
Unemployed	40	26.0
Retired	64	41.6
**Religious beliefs**		
No	142	92.2
Buddhism	4	2.6
other	8	5.2
**Family monthly income *per capita* (Chinese yuan/RMP)**		
<2000	13	8.4
2001~5,000	63	40.9
5,001~10,000	47	30.5
>10,000	31	20.1
**Medical insurance**		
Rural cooperative medical services	25	16.2
Medical insurance for urban residents	58	37.7
Out-of-town medical insurance	3	1.9
Employee medical insurance	66	42.9
Business insurance	2	1.3
**TNM stage**		
I	33	21.4
II	71	46.1
III	45	29.2
IV	5	3.2
**Time of illness**		
<3 months	51	33.1
3~6 months	55	35.7
6~12 months	36	23.4
1~3 years	3	1.9
3~5 years	5	3.2
>5 years	4	2.6
**Current or previous treatment modality**		
Surgery + radiotherapy	5	3.2
Surgery + chemotherapy	132	85.7
Surgery + chemotherapy + radiotherapy	17	11
**Transfer**		
Yes	25	16.2
No	129	83.8
**With or without children**		
Yes	144	93.5
No	10	6.5

### 4.2 Mean scores of body image, dyadic coping and PTG in breast cancer patients

The mean scores for body image, dyadic coping, and post-traumatic growth were 79.98 (17.29), 113.24 (20.79), and 62.33 (16.64), respectively. The mean scores for the other dimensions are shown in Table 2.

**Table 2:** Mean scores of variables and subcategories (*n* = 154).

Variables		Mean scores *M* (SD)
Body image		79.98 (17.29)
	Body image-related behavioral changes	24.34 (6.85)
	Body image-related sexual activity	13.73 (3.67)
	Body image-related role changes	13.29 (3.26)
	Body image-related psychological changes	22.87 (5.41)
	Body image-related social changes	5.75 (1.55)
Dyadic coping		113.24 (20.79)
	Stress communication	30.16 (7.38)
	Support coping	31.93 (9.41)
	Empowering coping	14.60 (3.52)
	Negative coping	20.01 (3.71)
	Common coping	16.55 (5.02)
Post-traumatic growth		62.33 (16.64)
	Relating to others	7.32 (2.36)
	New possibilities	12.36 (4.33)
	Personal strength	11.08 (3.29)
	Spiritual change	13.26 (3.46)
	Appreciation of life	18.31 (6.07)

## 5 Correlation analysis between body image, dyadic coping, and post-traumatic growth

Pearson correlation analyses of body image, dyadic coping, and post-traumatic growth showed that the level of body image was negatively correlated with post-traumatic growth (*r* = −0.462, *p* < 0.01). The level of body image was negatively correlated with dyadic coping (*r* = −0.308, *p* < 0.01). And dyadic coping was positively correlated with post-traumatic growth (*r* = 0.464, *p* < 0.01). The correlation analysis of other variables is shown in Table 3.

**Table 3:** Correlations between body image, dyadic coping and PTG (*n* = 154).

Variable	PTG	Relating to others	New possibilities	Personal strength	Spiritual change	Appreciation of life	Body image
Body image	−0.462^**^	−0.370^**^	−0.360^**^	−0.304^**^	−0.433^**^	−0.454^**^	–
Body image-related Behavioral changes	−0.279^**^	−0.342^**^	−0.337^**^	−0.478^**^	−0.431^**^	−0.452^**^	0.905^**^
Body image-related sexual activity	−0.315^**^	−0.253^**^	−0.229^**^	−0.184^*^	−0.259^**^	−0.355^**^	0.839^**^
Body image-related role changes	−0.437^**^	−0.335^**^	−0.355^**^	−0.258^**^	−0.383^**^	−0.457^**^	0.807^**^
Body image-related psychological changes	−0.353^**^	−0.392^**^	−0.266^**^	−0.246^**^	−0.338^**^	−0.299^**^	0.864^**^
Body image-related social changes	−0.257^**^	−0.222^**^	−0.291^**^	−0.064	−0.119	−0.308^**^	0.452^**^
Dyadic coping	0.464^**^	0.306^**^	0.370^**^	0.358^**^	0.379^**^	0.480^**^	−0.308^**^
Stress communication	0.384^**^	0.272^**^	0.308^**^	0.305^**^	0.299^**^	0.390^**^	−0.219^**^
Support coping	0.431^**^	0.286^**^	0.346^**^	0.313^**^	0.351^**^	0.454^**^	−0.323^**^
Empowering coping	0.435^**^	0.306^**^	0.357^**^	0.299^**^	0.393^**^	0.434^**^	−0.337^**^
Negative coping	−0.280^**^	−0.244^**^	−0.290^**^	−0.116	−0.196^*^	−0.291^**^	0.279^**^
Common coping	−0.453^**^	−0.296^**^	−0.395^**^	−0.324^**^	−0.342^**^	−0.473^**^	−0.318^**^

PTG, post-traumatic growth.*Significant at *p* < 0.05 level.**Significant at *p* < 0.01 level.

## 6 A test of the mediating effect of dyadic coping in the body image and post-traumatic growth in breast cancer patients

**Figure 2:**
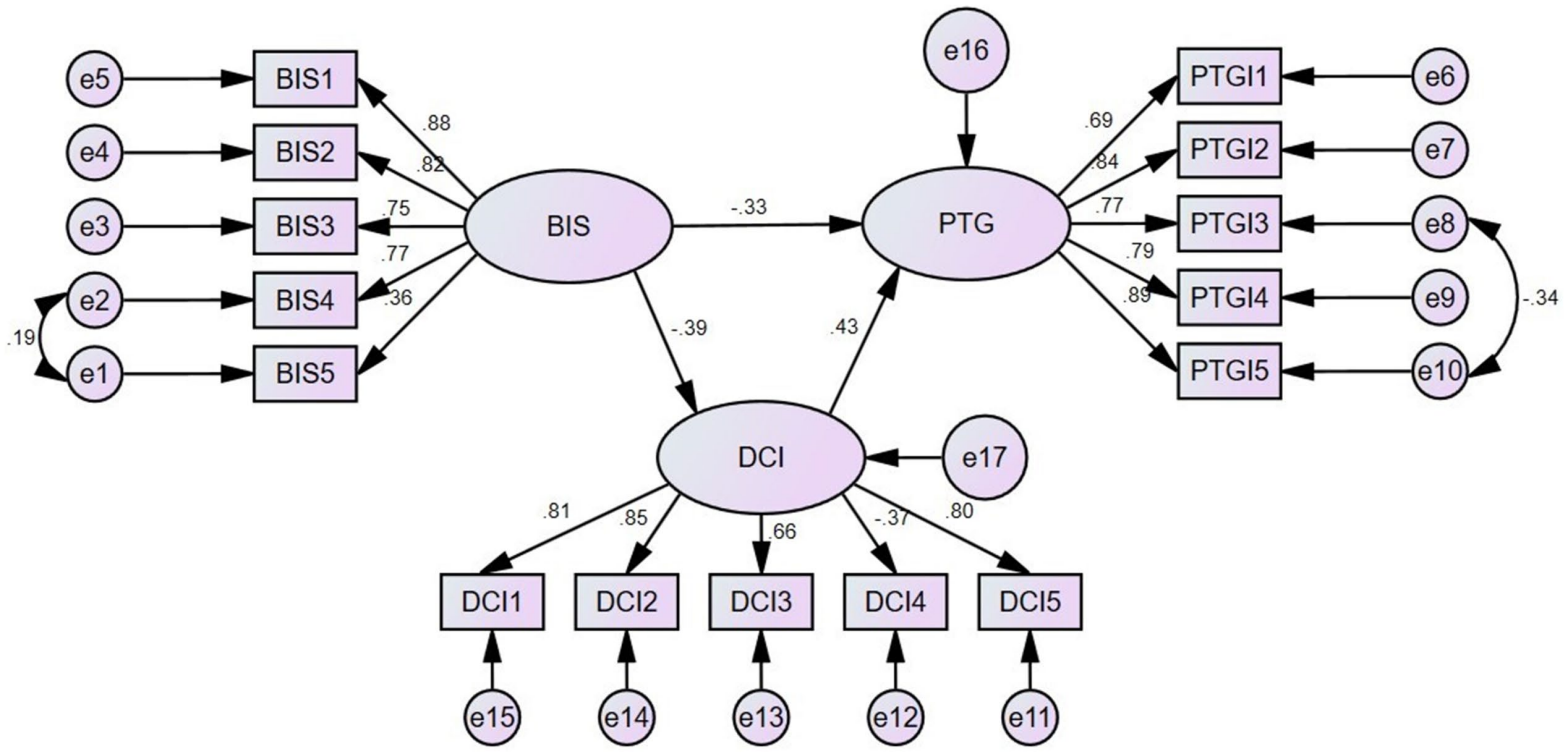
The structural equation model of body image, dyadic coping, and post-traumatic growth (PTG). BIS1: Body image-related behavioral changes. BIS2: Body image-related sexual activity. BIS3: Body image-related role changes. BIS4: Body image-related psychological changes. BIS5: Body image-related social changes. DCI1: Stress communication. DCI2: Support coping. DCI3: Empowering coping. DCI4: Negative coping. DCI5: Common coping. PTGI1: Relating to others. PTGI2: New possibilities. PTGI3: Personal strength. PTGI4: Spiritual change. PTGI5: Appreciation of life.

Based on the results of the correlation analysis, it can be seen that there is a two-by-two correlation between the body image, dyadic coping, and post-traumatic growth, with both the body image and dyadic coping significantly predicting post-traumatic growth, and the body image in turn significantly predicting dyadic coping. In order to further explore the role of the relationship between the three variables, this study used Amos software to conduct a mediation effect test, based on which a structural equation model was established to validate the mediating role of the dyadic coping between the body image and post-traumatic growth. The maximum likelihood method was used to estimate the model parameters, and the model was corrected according to the correction index, and the results showed that the fitting indexes in this study basically met the standards, as shown in Table 4. The structural equation model is shown in Figure 2.

**Table 4:** Fit indicators for mediated effects models.

Model fit	χ^2^/df	GFI	CFI	NFI	TLI	IFI	RMSEA
Fitness index	2.05	0.93	0.99	0.93	0.99	0.99	0.03
Standard value	<5	≥0.9	≥0.9	≥0.9	≥0.09	≥0.9	<0.08

χ^2^/df, The chi-square to degrees of freedom ratio; GFI, goodness-of-fit index; CFI, comparison of fit indices; NFI, canonical fit index; TLI, non-canonical fit index; IFI, incremental fit index; RMSEA, root mean square of approximate error difference.

## 7 Bootstrap mediation effects test

The structural equation modeling path coefficients showed that body image had a significant negative effect on dyadic coping (*β* = −0.39, *p* < 0.05). The body image had a significant negative effect on post-traumatic growth (*β* = −0.33, *p* < 0.05). And the body image indirectly impacted post-traumatic growth through dyadic coping (*β* = −0.17, *p* < 0.05) (see Table 5 for details).

**Table 5:** Path coefficient of structural model mediated by dyadic coping.

Path	Unstandardized effects	Standardized effects	SE	CR	*p*
BIS→DCI	−2.773	−0.390	0.877	−3.162	0.002
DCI → PTG	0.177	0.431	0.038	4.677	***
BIS→PTG	−0.968	−0.333	0.325	−2.977	0.003

BIS, body image disturbance; DCI, dyadic coping inventory; PTG, post-traumatic growth.

To further test whether dyadic coping mediates the relationship between body image and post-traumatic growth in breast cancer patients, the Bootstrap mediated effects test was used to examine the mediated effects results. The results showed that the direct effect of body image on post-traumatic growth was −0.968, with a 95% confidence interval [−2.070–0.475], not including 0. The direct effect was significant, meaning that the body image in breast cancer patients can directly influence post-traumatic growth. The mediating effect of dyadic coping was −0.489 with a 95% confidence interval of [−1.095–0.222], not including 0, indicating a partial mediating effect. The final total effect of dyadic coping mediation was −1.458, which means that dyadic coping partially mediated the effect between body image and post-traumatic growth, accounting for 34% of the total effect ratio. This is shown in Table 6.

**Table 6:** Path coefficient of standardized structural model mediated by dyadic coping.

Path	Estimate	SE	Bias-corrected 95%CI		*p*	Effect ratio
			Lower	Upper		
Mediating effect	−0.489	0.214	−1.095	−0.222	0.001	34%
Direct effect	−0.968	0.408	−2.070	−0.475	0.001	66%
Total effect	−1.458	0.510	−2.694	−0.860	0.001	**–**

## 8 Discussion

The primary purpose of this study was to examine the relationships between body image, dyadic coping, and post-traumatic growth, as well as the role of dyadic coping as a mediator in these relationships. The results of the study showed that the body image score of breast cancer patients was 79.98 (17.29), which is similar to the results of the study by An et al. (2018). It indicates that there is a status quo of low level of body image in breast cancer patients. The highest scoring dimension in this study was 24.34 (6.85) for body image-related behavioral changes, the changes in body image due to breast loss, hair loss and weight gain caused by the treatment received after surgery lead to lowered self-esteem, sensitivity and shame, etc., and therefore due to the fact that breast cancer patients are more concerned about their body image (Brunet et al., 2022). In daily life and in public places, patients are prone to behavioral changes and other ways of concealing or disguise their body defects to avoid embarrassment when others notice their body defects (Ettridge et al., 2022). Body image-related psychological change scores ranked second in the dimensions scores 22.87 (5.41), and previous studies have shown that breast cancer patients experience more severe negative psychological changes due to treatment-induced changes in appearance (Liu et al., 2022). Many patients perceive that the change in body image affects attitudes or feelings about their self-image, and are disappointed with their body image (Hoyle et al., 2022), and when faced with scarring in their surgical area they cannot help but associate it with the experience of the disease, even fearing that the disease may return at any time (Wu et al., 2021; Phoosuwan and Lundberg, 2023). Body image-related sexual activity scores ranked third in the dimensions scores 13.73 (3.67), and previous studies have shown that sexual activity is closely related to body image (Sebri et al., 2021). On the one hand, surgery-induced scarring or mastectomy can lead to a variety of psychosocial problems, including reduced attraction and satisfaction with the body, decreased self-esteem and self-efficacy, and can adversely affect the sexual experience (Yan et al., 2020). On the other hand, breasts are considered to be a symbol of femininity and image (Smedsland et al., 2022). Changes in body image associated with BC may cause them to feel dissatisfied with their bodies, believing that they have lost their original femininity, and thus become suspicious of their partners, wondering if they still have any value in the minds of their spouses, and leading to less initiation of sexual activity between partners (Velasco et al., 2023).

In this study, we found that the dyadic coping of breast cancer patients was at an average level with a score of 113.24 (20.79), which is lower than the findings of Shi et al. (2021). It may be due to the fact that most of the participants in our study belonged to the young and middle-aged age group, and young and middle-aged breast cancers do not communicate with their spouses in a very frequent manner compared to the older patients, and lack of communication about the stress to communicate with their spouses in a positive and effective way to cope (Varner et al., 2019). The results show that the empowering coping dimension has the lowest score of 14.60 (3.52), probably because the spouses of breast cancer patients play the role of the family breadwinner, where, on the one hand, the husband is required to bear all the financial expenses of the family, thus neglecting to help his wife in her daily life. On the other hand, the wife cannot work normally due to her illness and cannot help her husband to bear the financial burden of the family. This prevents the husband and wife from having a normal empowering coping.

Our study also found moderate levels of post-traumatic growth in breast cancer patients with a score of 62.33 (16.64), which is consistent with previous studies (Fu et al., 2022; Liu et al., 2022). In our study, breast cancer patients required adjuvant treatments such as chemotherapy and radiation after surgery, and it has been demonstrated that patients with more complex treatment modalities as well as longer treatment cycle durations suffered more psychological, physical, and financial burdens than those with a single treatment modality (Choi et al., 2022). As a result, post-traumatic growth disorders are subsequently more severe.

Finally, structural equation modeling indicated that the level of body image was negatively correlated with dyadic coping and post-traumatic growth; whereas dyadic coping was positively correlated with post-traumatic growth, which verified our Hypothesis 1. This suggests that patients with low levels of body image also have lower levels of dyadic coping and post-traumatic growth, which may be explained by the fact that breast cancer patients who report more body image problems may also face more challenges in coping with the disease (Michalczyk et al., 2022), and perceive that their partners engage in negative dyadic behaviors more frequently. Patients with a low level of body image are reluctant to share their thoughts and feelings with their husbands due to the development of negative psychological aspects such as low self-esteem and stigma, which creates a disconnect and suspicion between the two parties, thus impeding the dyadic behavior of the couple. Our findings also report that breast cancer patients who face lower levels of body image have more difficulty recovering from trauma, an outcome that may be detrimental due to the fact that patients are constantly exposed to the side effects of treatment and are constantly burdened with the financial stress and psychological burdens that come with treatment, thus discouraging patients from recovering from their traumas (Hsu et al., 2021). Previous studies of in-depth interviews with breast cancer patients who developed post-traumatic growth found that patients mentioned that they were not only able to look at their “new bodies” in front of the mirror and accept them, but also gave them a new meaning, i.e., the “new body” was a constant reminder of their mortality and the meaning of life (Su and Fang, 2018). This shows that improving the body image in breast cancer patients is an effective way to improve dyadic coping and post-traumatic growth. Structural equation modeling also suggests that dyadic coping has a positive effect on post-traumatic growth, i.e., the higher the dyadic coping scores of BC patients, the higher the level of post-traumatic growth. This may be related to the fact that the patient receives adequate support from his or her spouse’s verbal, behavioral, and perceptual messages.

As we expected, dyadic coping partially mediated the relationship between body image and post-traumatic growth. This verifies our Hypothesis 2. Improving dyadic coping in BC couples can indirectly promote post-traumatic growth by improving the patient’s body image. In other words, body image can have an impact on dyadic coping, which in turn can have an impact on post-traumatic growth.

Therefore, nurses and psychologists should emphasize and enhance the assessment of the body image in breast cancer patients (Fiser et al., 2021), it is also necessary to pay attention to the changes in behavioral, psychological, and sexual activities related to body image, and to have patients undergo targeted psychological interventions in a timely manner, which may help to promote post-traumatic growth by enhancing the dyadic coping between the patients and their spouses, and by decreasing the suspicion between the couples in order to enhance the patients’ self-confidence and reduce their low self-esteem and negative psychology.

## 9 Limitations

First, our study is a single-center study. Due to individual differences, our findings cannot be generalized to other cities in China. Future studies should include multicenter clinical investigations to increase sample diversity. Second, our study that we investigated only among breast cancer patients, so we cannot exclude that the significant shock suffered by their husbands as a result of their wives’ illnesses and their dyadic coping styles toward their wives could affect the body image of breast cancer patients. Therefore, future studies should include dyadic coping and post-traumatic growth in partners. Third, body image, dyadic coping, and post-traumatic growth in breast cancer patients can be adjusted according to the different stages of the disease; however, this cross-sectional study was unable to explore the dynamic trends among the three. In future studies, a longitudinal study should be designed to elucidate the dynamic trends of body image, dyadic coping, and post-traumatic growth. Finally, this was a cross-sectional design and our study was not able to assess causality between the study variables; future studies should design a longitudinal study examining causality of the variables.

## 10 Conclusion

Chinese BC patients had high levels of body image disturbance and moderate levels of post-traumatic growth. The level of body image was negatively correlated with dyadic coping and post-traumatic growth, whereas dyadic coping was positively correlated with post-traumatic growth. Dyadic coping plays an active role in promoting post-traumatic growth, significantly moderating the relationship between body image and post-traumatic growth. Therefore, in the nursing process of breast cancer patients, on the one hand, close attention should be paid to the coping styles of the patients and their spouses, so that the patients and their spouses can be regarded as a community of disease, encouraged to adopt a positive way to face the trauma, and their dyadic coping can be improved. On the other hand, there is a need to strengthen the assessment of the patient’s body image, and healthcare professionals can improve the patient’s body image and dyadic coping through the development of targeted interventions, which will in turn promote post-traumatic growth.

## Data availability statement

The original contributions presented in the study are included in the article/supplementary material, further inquiries can be directed to the corresponding authors.

## Ethics statement

The studies involving humans were approved by this study was approved by the Medical Ethics Committee of the Affiliated Hospital of Jiangnan University, Wuxi City, Jiangsu Province, China (JNMU020423785). The studies were conducted in accordance with the local legislation and institutional requirements. The participants provided their written informed consent to participate in this study. Written informed consent was obtained from the individual(s) for the publication of any potentially identifiable images or data included in this article.

## Author contributions

YWa: Data curation, Formal analysis, Investigation, Methodology, Software, Visualization, Writing – original draft. SW: Data curation, Formal analysis, Investigation, Writing – original draft. LT: Writing – original draft, Data curation, Investigation. JZ: Data curation, Investigation, Writing – original draft. YX: Data curation, Investigation, Writing – original draft. YWu: Writing – original draft, Conceptualization, Funding acquisition, Supervision, Writing – review & editing. LC: Conceptualization, Funding acquisition, Supervision, Validation, Writing – review & editing, Writing – original draft.
